# BML-111 inhibit H_2_O_2_-induced pyroptosis and osteogenic dysfunction of human periodontal ligament fibroblasts by activating the Nrf2/HO-1 pathway

**DOI:** 10.1186/s12903-023-03827-w

**Published:** 2024-01-08

**Authors:** Yao Xu, Yi Chu, Wanrong Yang, Kefei Chu, Sihui Li, Ling Guo

**Affiliations:** 1 Luzhou Key Laboratory of Oral and Maxillofacial Reconstruction and Regeneration, The Affiliated Stomatological Hospital of Southwest Medical University, Luzhou, China; 2 Department of Oral prosthodontics, The Affiliated Stomatological Hospital of Southwest Medical University, Luzhou, China; 3 The people’s hospital of pengzhou, Chengdu, China

**Keywords:** Lipoxin A4 analog, Reactive oxygen species, NLRP3, Pyroptosis, Nrf2

## Abstract

**Background:**

Periodontitis is a common and harmful chronic inflammatory oral disease, characterized by the destruction of periodontal soft and hard tissues. The NLRP3 inflammasome-related pyroptosis and human periodontal ligament fibroblasts (hPDLFs) osteogenic dysfunction are involved in its pathogenesis. Studies have shown that lipoxin A4 is an endogenous anti-inflammatory mediator and BML-111 is a lipoxin A4 analog, which was found to have potent and durable anti-inflammatory effects in inflammatory diseases, but the mechanism remains unclear. The purpose of this study was to investigate whether BML-111 inhibits H_2_O_2_-induced dysfunction of hPDLFs, attenuates inflammatory responses, and identifies the underlying mechanisms.

**Methods:**

The oxidative stress model was established with H_2_O_2_, and the cell proliferation activity was measured by CCK-8. ALP staining and alizarin red staining were used to detect the osteogenic differentiation capacity of cells; flow cytometry and ELISA were used to detect cell pyroptosis; we explored the effect of BML-111 on hPDLFs under oxidative stress by analyzing the results of PCR and Western blotting. The Nrf2 inhibitor ML385 was added to further identify the target of BML-111 and clarify its mechanism.

**Results:**

BML-111 can alleviate the impaired cell proliferation viability induced by H_2_O_2_. H_2_O_2_ treatment can induce NLRP3 inflammasome-related pyroptosis, impairing the osteogenic differentiation capacity of hPDLFs. BML-111 can effectively alleviate H_2_O_2_-induced cellular dysfunction by activating the Nrf2/HO-1 signaling pathway.

**Conclusion:**

The results of this study confirmed the beneficial effects of BML-111 on H_2_O_2_-induced NLRP3 inflammasome-related pyroptosis in hPDLFs, and BML-111 could effectively attenuate the impaired osteogenic differentiation function. This beneficial effect is achieved by activating the Nrf2/HO-1 signaling pathway, therefore, our results suggest that BML-111 is a potential drug for the treatment of periodontitis.

**Supplementary Information:**

The online version contains supplementary material available at 10.1186/s12903-023-03827-w.

## Introduction

Periodontitis is one of the most common chronic infectious oral diseases and is the main cause of tooth loss in adults. Uncontrolled periodontitis can lead to the continuous destruction of soft and hard periodontal supporting tissues, impairing one’s chewing and aesthetic functions [1]. In addition, severe periodontitis is a contributing factor to systemic diseases such as atherosclerosis and diabetes, which damage a person’s general health. Finding an effectively strategy to control periodontitis and promote the regenerative repair of periodontal tissues is a hot research topic in recent years. Human periodontal fibroblasts (hPDLFs) are the most abundant cells with the potential of multi-directional differentiation in the periodontal ligament tissue. The literature shows that they have osteogenic differentiation ability and contribute to the regeneration of alveolar bone [2]. However, inflammation and oxidative stress can lead to osteogenic dysfunction of hPDLFs [3]. Hence, in recent years, how to prevent hPDLFs from dysfunction under different stress conditions and preserve their osteogenic differentiation capacity has become a new strategy for the treatment of periodontitis.

Previous studies have shown that periodontal pathogens are the main causes of periodontitis, and the host immune responses caused by periodontal pathogens and injury factors also play an important role in the pathogenesis of periodontitis [4]. Reactive Oxygen Species (ROS), produced by immune cells, is thought to be a key factor in the development of periodontitis. The overproduced ROS in periodontal tissue activated the NF-κB signaling pathway and NLRP3 inflammasome, participating the inflammatory process [5, 6]. Studies have shown that activated NLRP3 inflammasome can further activate Caspase-1, leading to the maturation of IL-1β and IL-18, as well as the cleavage of GSDMD protein, which resulting in cell pyroptosis and inflammation [7]. Besides, the activated NLRP3 inflammasome can also intrigue cell autophagy, promote the differentiation of osteoclasts and inhibit the osteogenic function of hPDLFs, finally promoting the periodontitis [8]. The cytoprotective gene Nrf2 (nuclear factor-erythropoietin 2-related factor 2) is a redox-sensitive leucine zipper transcription factor with antioxidant and anti-inflammatory properties [9].Under static conditions, Nrf2 is inactive binding to Keap1, but when oxidative stress occurs, Nrf2 dissociates from Keap1 and transports from the cytoplasm to the nucleus, where it plays a role in activating the antioxidant element ARE, and then increases the expression of antioxidant enzymes HO-1 and NQO-1 [10]. Thus, the dissociation of Nrf2-Keap1 complex helps removing the overproduced ROS under oxidative stress and inflammation to defend against oxidative stress and inflammatory damage [11]. Researchers found that activating Nrf2/HO-1 signal pathway inhibited NLRP3 inflammation and pyroptosis [12].

Lipoxin (LX) is an endogenous metabolite produced by arachidonic acid through different lipoxygenase catalysis under the stimulation of inflammatory factors, which mainly plays a biological role in regulating inflammation [13]. Lipoxin A4 (LXA4), one of the lipoxins, plays an important role in the inflammatory processes such as ischemia reperfusion injury, peritonitis, gastroenteritis and keratitis, and is it had been shown that Lipoxin A4 could up-regulate the expression of P62, competing with Nrf2 to bind the keap1 protein, activating the Nrf2 pathway and ameliorating cell damage [14]. Besides, studies have shown that lipoxin A4 participates in the regulation of bacterial biofilm and cell microenvironment, inhibit or eliminate pathogens, and maintain the homeostasis of local internal environment during the resolution of periodontal inflammation [15–18]. However, LXA4 is characterized by rapid inactivation and short half-life, which limits its clinical application. BML-111, as a stable and long-acting LXA4 analogue, can activate FPR2/ALXR receptors and exert similar anti-inflammatory effects comparable to LXA4 in several pathological in vivo models [19]. BML-111 has been shown to play a pro-resolution role in inflammatory models such as acute lung injury, collagenous arthritis, and acute pancreatitis by activating the Nrf2/HO-1 pathway and inhibiting the NF-κB/NLRP3 signaling pathway [20], but its role in periodontitis remains unclear.

Therefore, the aim of this study is to establish a NLRP3 inflammasome-related pyroptosis model by H_2_O_2_ to increase intracellular ROS content. HPDLFs were treated with different concentrations of BML-111 to observe the changes in intracellular ROS, pyroptosis, and osteogenic differentiation. To investigate the regulatory mechanism of BML-111 on H_2_O_2_-induced pyroptosis and osteogenic differentiation dysfunction in hPDLFs by introducing the Nrf2 inhibitor ML385, and to provide laboratory and theoretical support for the prevention and treatment of periodontitis.

## Materials and methods

### Materials and reagents

BML-111 was acquisited from Cayman (USA). Nrf2 inhibitor ML385 was purchased from MedChemExpress (Shanghai, China). H_2_O_2_ was accessed from Solarbio (Beijing, China). The antibodies against ALP, OCN, RUNX-2, NLRP3, ASC, Caspase-1, Nrf2, HO-1, P62, Keap1, Lamin A/C and GAPDH were purchased from Bioswamp (Wuhan, China). Antibodies against GSDMD-N were purchased from Abcam (Cambridge, MA, USA).

### Cell culture

HPDLFs were obtained from BeNa Culture Collection (Henan, China), which identified by Cell Line Authentication by STR profiling. The cells were cultured in the complete medium [a-minimum essential medium (Gibco, CA, United States), 10% fetal bovine serum (Sijiqing, Zhejiang, China), 1% penicillin-streptomycin].

### Cell viability assay

To detect cell proliferation viability and obtain optimal drug concentrations of H_2_O_2_ and BML-111, we carried out the CCK8 assay (Beyotime, shanghai, China). HPDLFs were seeded into 96-well plates (5 × 10^3^ cells/well), and after culturing for 24 h, they were treated with 0, 125, 250, 500, 1000 µM H_2_O_2_ for 4 h. The second part was treated with BML-111 (0, 5, 10, 20, 30 µM) for 24 h, besides, the third part was treated with BML-111 (0, 5, 10, 20 µM) for 24 h and 500 µM H_2_O_2_ stimulation for 4 h. After drug pretreatment, CCK-8 reagent was adding to the cells, after incubation for 2 h, use a microplate reader (Thermo Fisher Scientific, Inc) to detect the OD value of each well at the absorbance of 450 nm. Each group was set up with 3 duplicate holes, and each experiment was repeated three times.

### Detect ROS generation

To detect intracellular ROS production, we chose the DCFH-DA probe (Beyotime, Shanghai, China) in this study. Firstly, hPDLFs were seeded in 6-well plates (1 × 10^5^ cells/well) and processed according to different groups. Add DCFH-DA (10 µM) to each well. The green fluorescence of ROS was observed by a fluorescence microscope (Leica, Germany) in the dark at room temperature. In addition, cells were collected and analyzed for fluorescence intensity by flow cytometry and FlowJo® software (BD Biosciences, USA). Each group was set up with 3 duplicate holes, and each experiment was repeated three times.

### Lactate dehydrogenase (LDH) release assay

LDH is normally located intracellularly and can be released from the cell into the extracellular space when pyroptosis occurs. To assess the effect of H_2_O_2_ on pyroptosis and the efficacy of BML-111, we detect LDH release with the LDH Assay Kit (Beyotime, Shanghai, China). HPDLFs were seeded in 96-well plate (3000/well). Cell supernatants were collected after treatment of different groups of cells, and the release of LDH was detected by LDH assay kit. Each group was set up with 3 duplicate holes, and each experiment was repeated three times.

### Detection of pyroptotic cells

HPDLFs were seeded in 6-well plates (1 × 10^6^ cells/well). After the cells adhered, they were treated with drugs according to different groups, then trypsinized, centrifuged, and resuspended in PBS. 2 × 10^4^ cells were selected and labeled with Annexin V-FITC and propidium iodide (PI). The detection was performed by flow cytometry within one hour, and the change in the percentage of cells in the double-positive-stained area was calculated. Each group was set up with 3 duplicate holes, and each experiment was repeated three times.

### Detection of osteogenic differentiation capacity

5 × 10^4^ hPDLFs were seeded into 12-well plates. When the density reached 70%, the hPDLFs were cultured in osteogenic differentiation medium for 7 days or 21 days, and the hPDLFs were treated in different groups every 2 days. Cells were fixed with 4% PFA (Biosharp, Beijing, China) and stained with BCIP/NBT ALP kit (Beyotime, Shanghai, China) after 7 days of medication. After 21 days of medication, cells were fixed with 4% PFA and stained with 2% Alizarin Red (Solaribo, Beijing, China). Quantify and process data using ImageJ. Each group was set up with 3 duplicate holes, and each experiment was repeated three times.

### RNA isolation and quantitative real-time polymerase chain reaction (qRT-PCR)

HPDLFs were seeded in 6-well plates (1 × 10^6^ cells/well). The expression of hPDLFs genes NLRP3, ASC, Caspase-1, HO-1, ALP, runx2, OCN was detected by qRT-PCR. Total RNA was extracted after drug treatment using Trizol (Ambion, USA) and reverse transcribed into cDNA using PrimeScript II RTase (TaKaRa, Japan). The real-time quantitative PCR was performed using KAPA Biosystems SYBR FAST qPCR Master Mix (USA). The relative gene level was determined by 2^−ΔΔCt^ method and standardized by GAPDH values. Table 1 shows the primer sequences used for RT-qPCR analysis. Each group was set up with 3 duplicate holes, and each experiment was repeated three times.

**Table 1:** Primers used for RT-qPCR

Gene	Forward Primer	Reverse Primer
GAPDH	GGGAAACTGTGGCGTGAT	GAGTGGGTGTCGCTGTTGA
NLRP3	TCGGAGATTGTGGTTGGG	GGGCGTTGTCACTCAGGT
ASC	TGGACGCCTTGGACCTC	CTTCCGCATCTTGCTTGG
Caspase-1	ATGGGCTCTGTTTTTATTGG	TGTCCTGGGAAGAGGTAGAA
HO-1	GCAGAGGGTGATAGAAGAGG	GTAAGGACCCATCGGAGAA
ALP	ATACAAGCACTCCCACTTCATC	TCTGCCTCCTTCCACCAG
OCN	GCAGCGAGGTAGTGAAG	TGAAAGCCGATGTGGT
RUNX2	TGGACGAGGCAAGAGT	GAGGCGGTCAGAGAAC

### Western blot analysis

HPDLFs were seeded in 6-well plates (1 × 10^6^ cells/well). Cells were harvested after treatment in different groups and lysed in RIPA lysis buffer (Solarbio, Beijing, China). Bicinchoninic acid assay kit (Solarbio, Beijing, China) was used to evaluate protein concentration. The proteins were separated by SDS-PAGE and transferred to PVDF membranes (Millipore, MA). The membranes were incubated with primary antibodies against ALP, RUNX-2, OCN, NLRP3, ASC, Caspase-1, GSDMD-N, Nrf2, HO-1, Keap1, P62, Lamin, and GAPDH at 4 °C overnight after being blocked for one hour in 5% nonfat milk in TBST. After TBST washes, PVDF membranes were incubated with secondary antibodies for 1 h at room temperature. Subsequently, membrane-bound antibody was measured by enhanced chemiluminescence (ECL) reagent (Millipore, MA) and then quantified by TANON GIS software. Each group was set up with 3 duplicate holes, and each experiment was repeated three times.

### Detection of pyroptosis-related cytokines by ELISA

HPDLFs were seeded in 6-well plates (1 × 10^6^ cells/well). After culturing for 24 h, drug treatment was performed according to different groups. The cell culture supernatants of each group were collected and centrifuged. And then the levels of IL-1β and IL-18 in cell culture supernatants were assessed by relevant ELISA kits (Bioswamp, Wuhan, China). Each group was set up with 3 duplicate holes, and each experiment was repeated three times.

### Statistical analysis

All the data were analyzed by Graphpad Prism 8 software. The one-way ANOVA was used to compare multiple groups, followed with Tukey’s post hoc test pairwise comparisons. The results were presented as mean ± SD. P < 0.05 was considered to be statistically significant.

## Results

### BML-111 alleviated the inhibition of proliferation of hPDLFs by H_2_O_2_

**Fig. 1:**
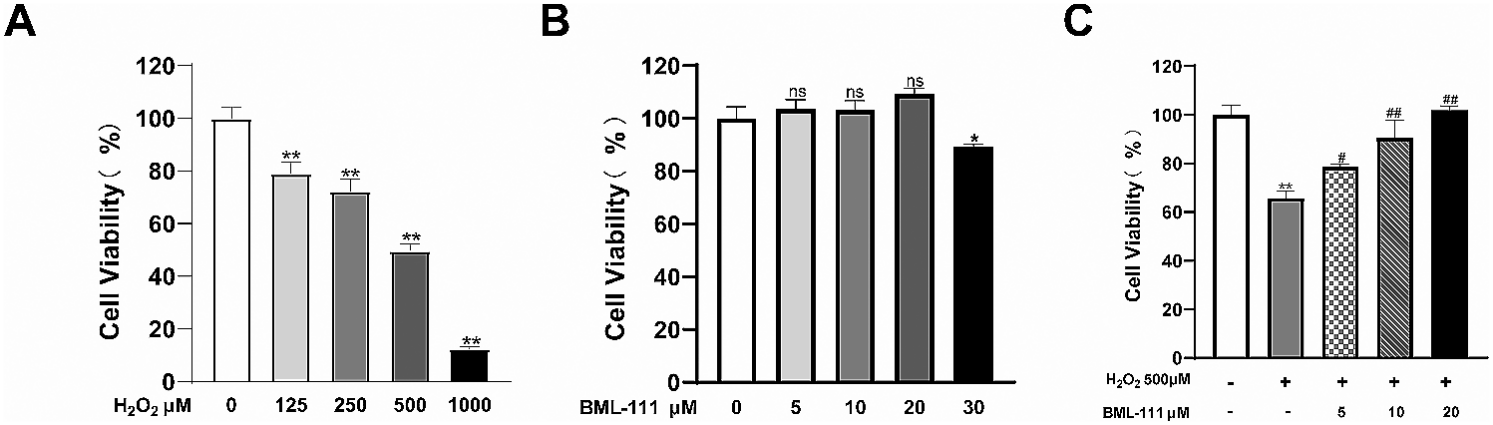
Effects of different concentrations of BML-111 and H_2_O_2_ on the cell viability of hPDLFs. **(A)** Cell viability of hPDLFs treated with 0, 125, 250, 500, 1000 µM H_2_O_2_ for 4 h; **(B)** Cell viability of hPDLFs treated with 0, 5, 10, 20, 30 µM BML-111 for 24 h; **(C)** Cell viability of hPDLFs pretreated with 0, 5, 10, 20 µM BML-111 for 24 h and treated with 500 µM H_2_O_2_ for 4 h. Data were shown as mean ± SD from three experiments independently ^**^P < 0.01 vs. control group, ^*^P < 0.05 vs. control group, ^##^*P* < 0.01 vs. H_2_O_2_ group, ^#^*P* < 0.05 vs. H_2_O_2_ group, ^ns^*P*>0.05 vs. control group

To investigate the protective effects of BML-111 against H_2_O_2_ treatment, cell viability was measured using the CCK-8 colorimetric assay. As showed in Fig. 1A, when the H_2_O_2_ concentration was greater than 125 µM, the proliferation activity of hPDLFs was decreased, and showed a concentration-dependent trend. When the cells were treated with 500 µM H_2_O_2_, the cell viability decreased about 50% compared with the control group. When the cells were treated by BML-111 with different concentrations (0, 5, 10, 20 µM), the cell proliferation activity gradually increased compared with control group, but decreased when the concentration of BML-111 was 30 µM (Fig. 1B). Different concentrations (0, 5, 10, 20 µM) of BML-111 were used to pretreat cells, and then cells were injured with 500 µM H_2_O_2_, the proliferation activity of cells in the BML-111 pretreatment group was gradually increased compared with the H_2_O_2_ group, and showed a concentration-dependent trend (Fig. 1C).

### BML-111 alleviated the increased intracellular ROS, pyroptosis rate and the release of inflammatory mediators in hPDLFs caused by H_2_O_2_

**Fig. 2:**
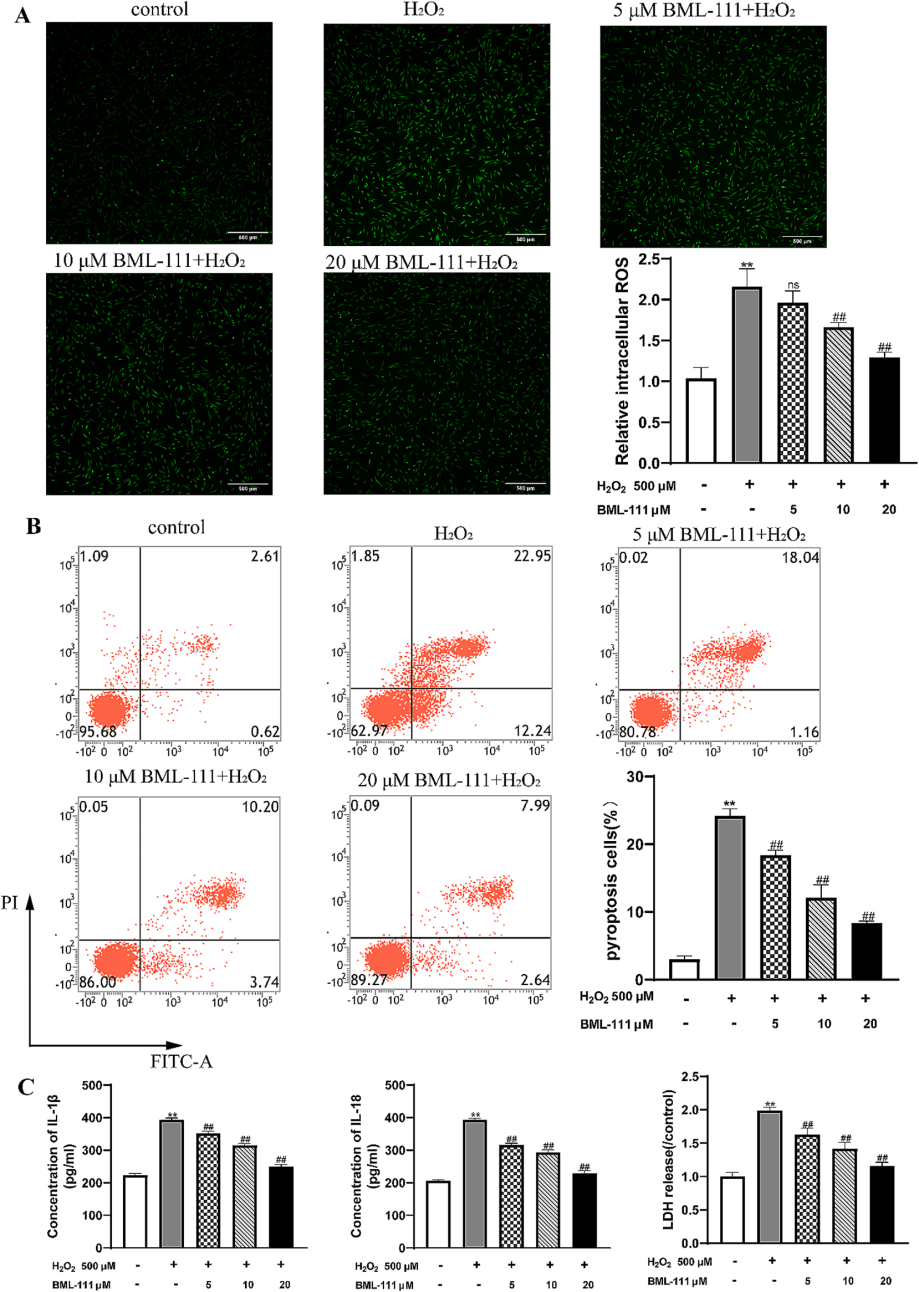
BML-111 suppressed H_2_O_2_-induced pyroptosis in hPDLFs. **(A)** Detection of intracellular ROS in hPDLFs by fluorescence staining and flow cytometry. **(B)** Flow cytometry detection of propidium iodide and annexin V fluorescein isothiocyanate (FITC) double-positive cells in hPDLFs. **(C)** the release of IL-1β, IL-18 and LDH from hPDLFs ^**^P < 0.01 vs. control group, ^##^*P* < 0.01 vs. H_2_O_2_ group, ^ns^*P*>0.05 vs. control group

As noted above, the increase of intracellular ROS activated the NLRP3 inflammasome in hPDLFs, which leads to the occurrence of pyroptosis. Pyroptosis is dependent on the activation of Caspase-1, followed by GSDMD protein cleavage, which is ultimately manifested in the release of IL-1β, IL-18, and LDH. As shown in Fig. 2A, when hPDLFs were treated with H_2_O_2_, intracellular ROS increased. Besides, the pyroptosis rate and the release of IL-1β, IL-18 and LDH were increased in H_2_O_2_-treated cells compared with the control group (Fig. 2B and C). However, BML-111 treatment decreased the intracellular ROS of hPDLFs treated with H_2_O_2_, and the pyroptosis rate and IL-1β, IL-18, LDH release were also decreased.

### BML-111 alleviated the osteogenic dysfunction of hPDLFs caused by H_2_O_2_

**Fig. 3:**
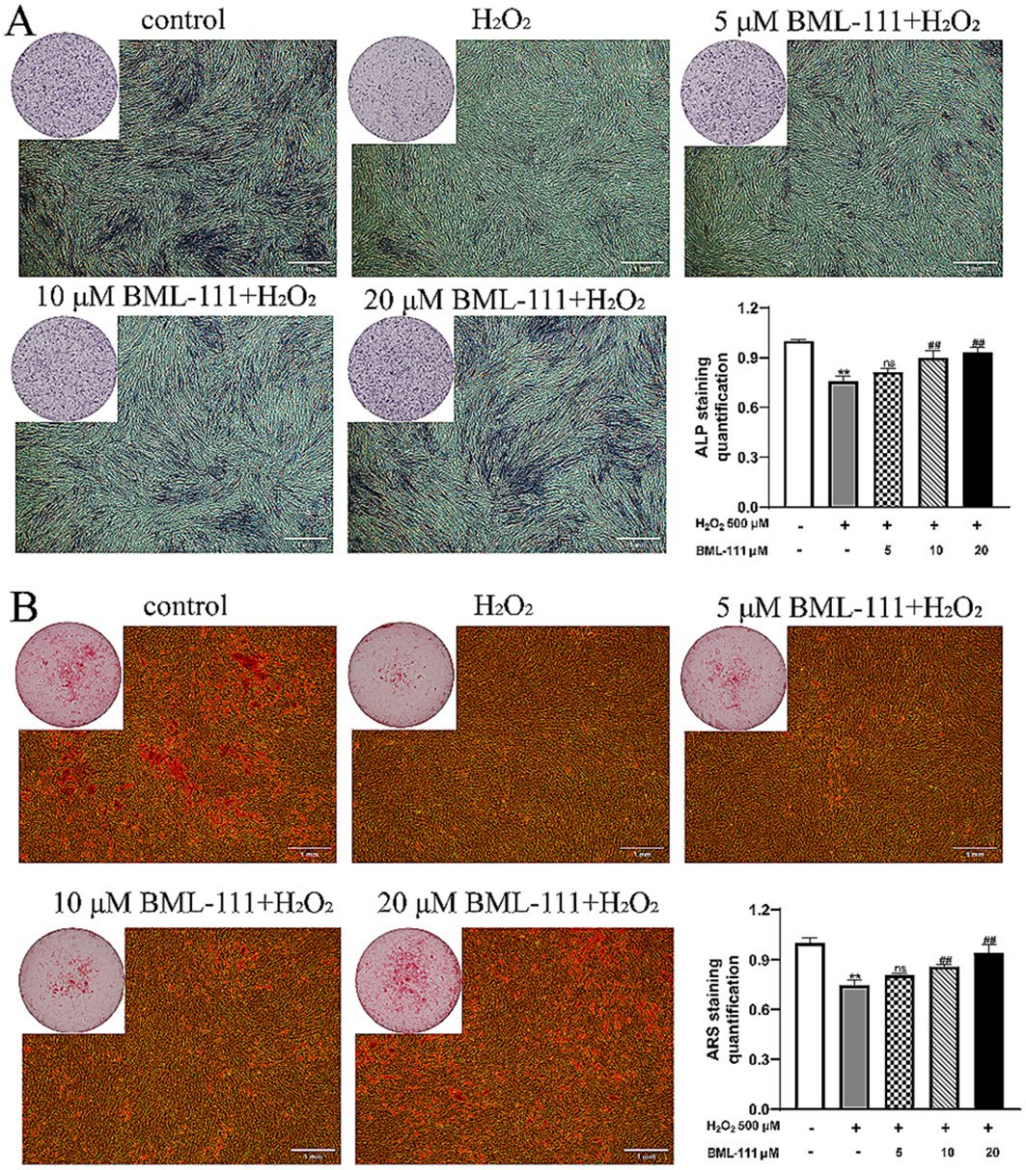
BML-111 mitigate H_2_O_2_-induced osteogenic dysfunction of hPDLFs. **(A)** Macroscopic and Microscopic images of ALP Staining and the quantification of hPDLFs. **(B)** Macroscopic and Microscopic images of Alizarin Red Staining and the quantification of hPDLFs. ^**^P < 0.01 vs. control group, ^##^*P* < 0.01 vs. H_2_O_2_ group, ^ns^*P*>0.05 vs. H_2_O_2_ group

HPDLFs have the ability to differentiate into osteoblasts. In this study, ALP staining, alizarin red staining was used to detect the effects of different treatments on the osteogenic differentiation ability of hPDLFs. As shown in Fig. 3, ALP secreted by hPDLFs on day 7 was stained blue, and calcium nodules produced on day 21 were stained orange-red by Alizarin red. The results showed that H_2_O_2_ treatment reduced the expression of ALP and the generation of calcium nodules in hPDLFs compared with the control group, while pretreatment with BML-111 could partially alleviate the damage of H_2_O_2_ on the osteogenic ability of hPDLFs (Fig. 3A and B).

### BML-111 activated the Nrf2/HO-1 pathway protected hPDLFs from H_2_O_2_-induced NLRP3 inflammasome related-pyroptosis

**Fig. 4:**
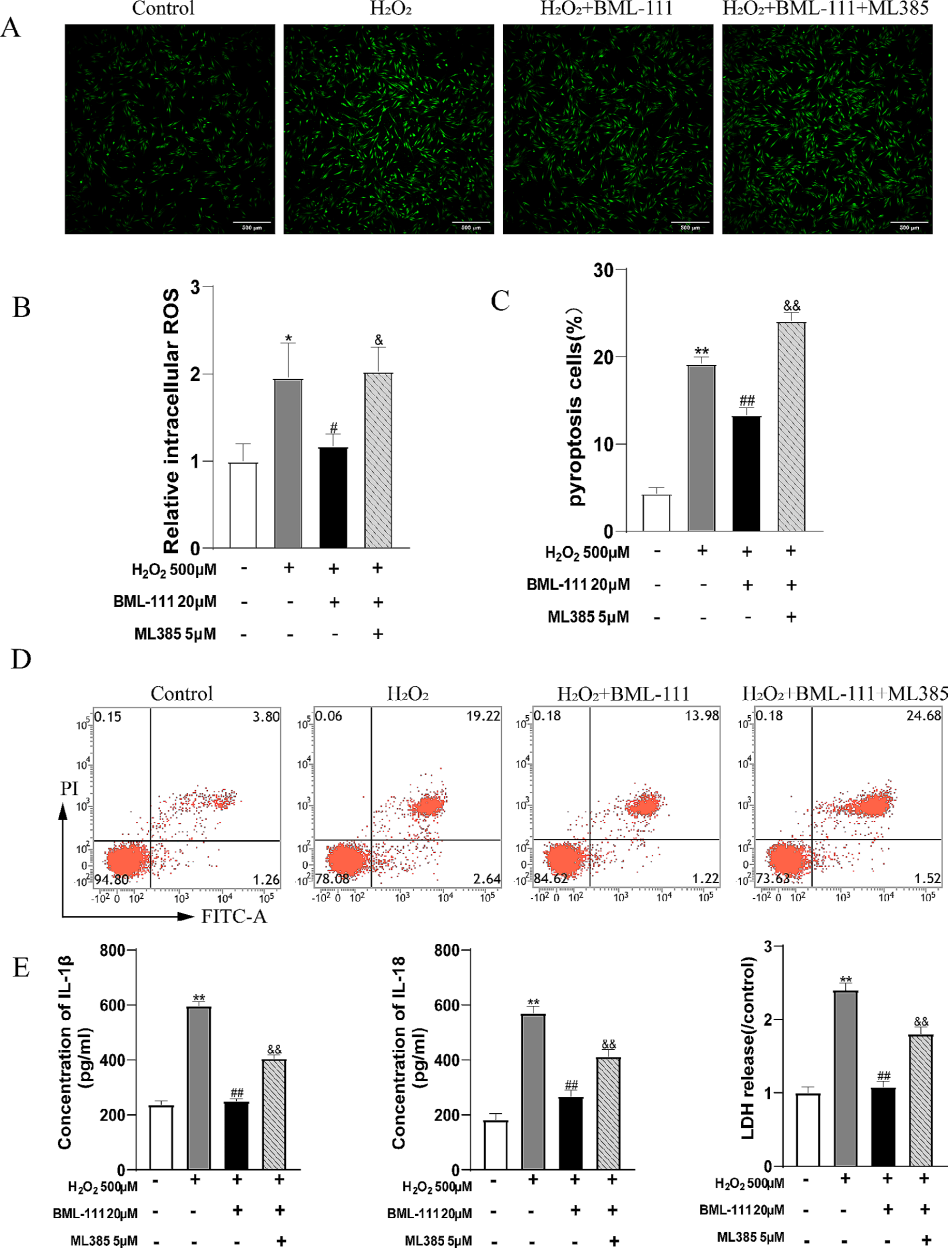
ML385 inhibit the effect of BML-111 on H_2_O_2_-induced pyroptosis in hPDLFs. Grouped as control, H_2_O_2_, H_2_O_2_ + BML-111, H_2_O_2_ + BML-111 + ML385 **(A)** Detection of intracellular ROS in hPDLFs by fluorescence staining. **(B)** The relative intracellular ROS in hPDLFs. **(C)** The pyroptosis rate of hPDLFs. **(D)** Flow cytometry detection of propidium iodide and annexin V fluorescein isothiocyanate (FITC) double-positive cells in hPDLFs. **(E)** The release of IL-1β, IL-18 and LDH from hPDLFs. ^**^P < 0.01 compared with control group. ^##^*P* < 0.01 compared with H_2_O_2_ group. ^&&^*P* < 0.01 compared with H_2_O_2_ + BML-111 group. ^*^P < 0.05 compared with control group. ^#^*P* < 0.05 compared with H_2_O_2_ group. ^&^*P* < 0.05 compared with H_2_O_2_ + BML-111 group

**Fig. 5:**
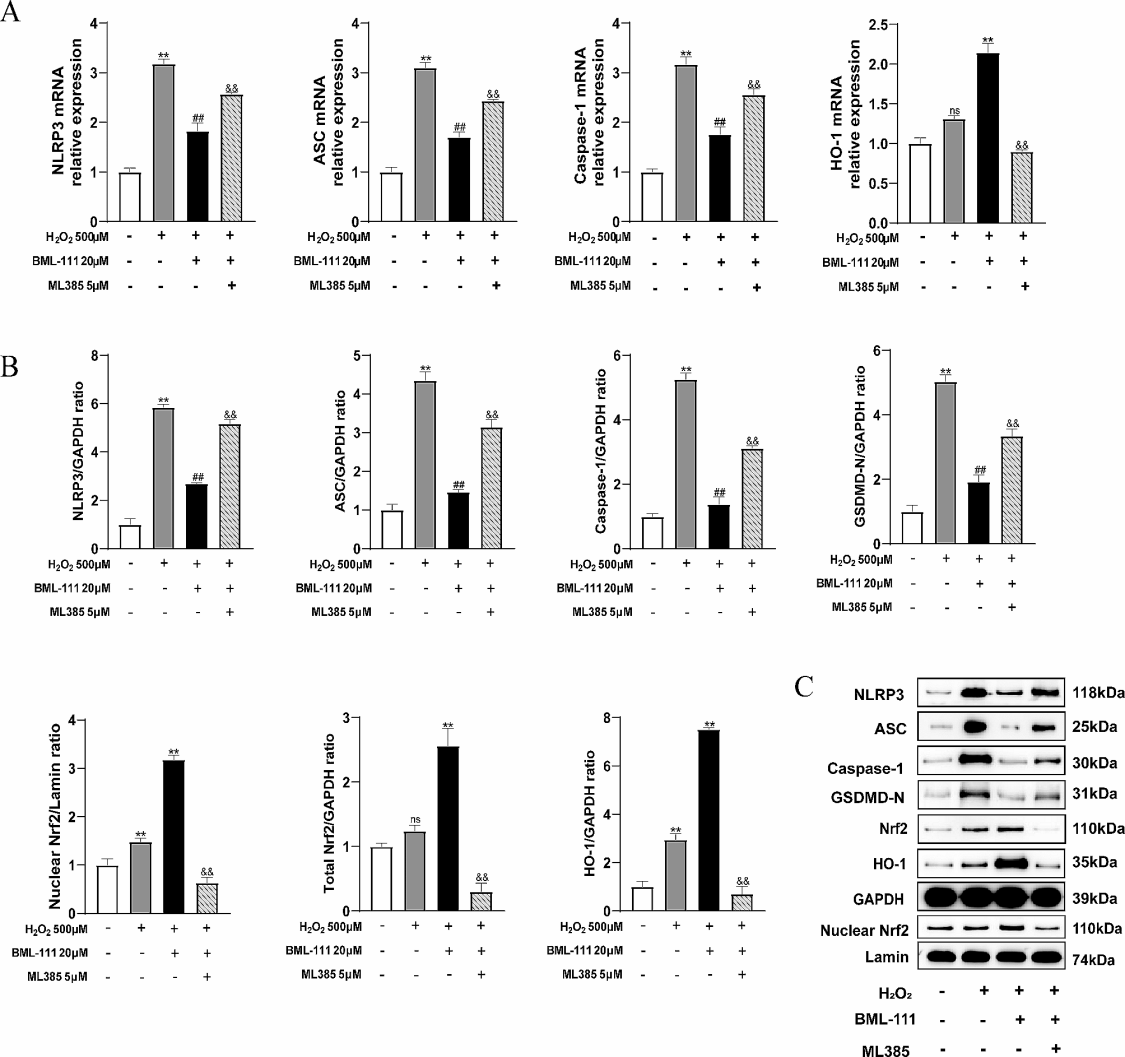
BML-111 reduced the expression of pyroptosis-related genes and proteins, and increased the expression of Nrf2 and HO-1 proteins in hPDLFs **(A)** The NLRP3, ASC, Caspase-1, HO-1 mRNA relative expression was shown. **(B)** The NLRP3, ASC, Caspase-1, GSDMD-N, Total Nrf2, Nuclear Nrf2, HO-1 protein relative expression was shown. **(C)** Representative Western blots for NLRP3, ASC, Caspase-1, GSDMD-N, Total Nrf2, Nuclear Nrf2, HO-1 protein. ^**^P < 0.01 compared with control group. ^##^*P* < 0.01 compared with H_2_O_2_ group. ^&&^*P* < 0.01 compared with H_2_O_2_ + BML-111 group. ^ns^*P*>0.05 compared with control group

**Fig. 6:**
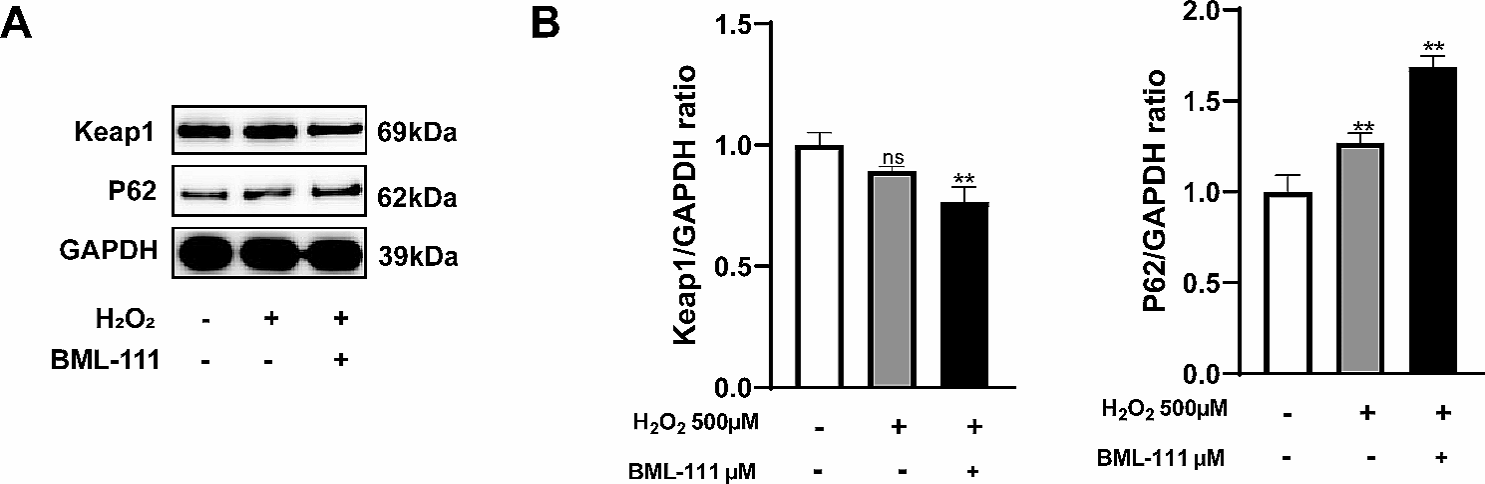
BML-111 reduced the expression of Keap1 proteins, and increased the expression of P62 protein in hPDLFs. **(A)** Representative blot for Keap1 and P62 protein. **(B)** The Keap1 and P62 protein relative expression was shown. ^**^P < 0.01 compared with control group. ^ns^*P*>0.05 compared with control group

Under oxidative stress, the overproduced ROS intrigued the NLRP3 inflammasome activation and resulted in pyroptosis. Besides, the intracellular antioxidant processes are initiated, including the activation of the Nrf2/HO-1 signaling pathway. As shown in Fig. 4A, when hPDLFs were treated with H_2_O_2_, intracellular ROS increased. Besides, the pyroptosis rate and the release of IL-1β, IL-18 and LDH were increased in H_2_O_2_-treated cells compared with the control group. However, BML-111 treatment decreased the intracellular ROS of hPDLFs treated with H_2_O_2_, and the pyroptosis rate and IL-1β, IL-18, LDH release were also decreased (Fig. 4B, C, D and E). The addition of ML385 inhibited the effect of BML-111 on H_2_O_2_-induced hPDLFs pyroptosis. As showed in Fig. 5A, the expression of NLRP3, ASC, Caspase-1 genes and the expression of NLRP3, ASC, Caspase 1, GSDMD-N proteins were significantly increased in H_2_O_2_ group compared with the control group (Fig. 5B). BML-111 effectively inhibited the expression of NLRP3, ASC, Caspase-1 and GSDMD-N induced by H_2_O_2_ (Fig. 5A and B), but the appliance of ML385 counteracted the effect of BML-111. Besides, the protein expression of Nrf2 in the H_2_O_2_ group was slightly increased than the control group, and the gene expression of HO-1 was also slightly increased (Fig. 5B). But the total protein expression of Nrf2 and the expression of nucleus Nrf2 protein of the BML-111 + H_2_O_2_ group were significantly increased than the H_2_O_2_ group, as also the gene expression levels of HO-1 (Fig. 5A). In order to test whether BML-111 treatment protects hPDLFs by activating the Nrf2 pathway in our experiments, we used an inhibitor of Nrf2(ML385). The results showed that ML385 + BML-111 + H_2_O_2_ group compared with the BML-111 + H_2_O_2_ group, the total amount of Nrf2 protein expression and the expression of nucleus Nrf2 protein of hPDLFs were significantly lower (Fig. 5C). As shown in the Fig. 6A and B, the protein expression of Keap1 in the H_2_O_2_ group was slightly decreased than the control group, and the application of BML-111 with H_2_O_2_ made the expression of Keap1 decreased than the H_2_O_2_ group. Nevertheless, the protein expression of P62 in the H_2_O_2_ group was slightly increased than the control group, and the expression of P62 protein in the BML-111 + H_2_O_2_ group were significantly increased than the H_2_O_2_ group.

### BML-111 activated the Nrf2/HO-1 pathway protected hPDLFs from H_2_O_2_-induced osteogenic dysfunction

**Fig. 7:**
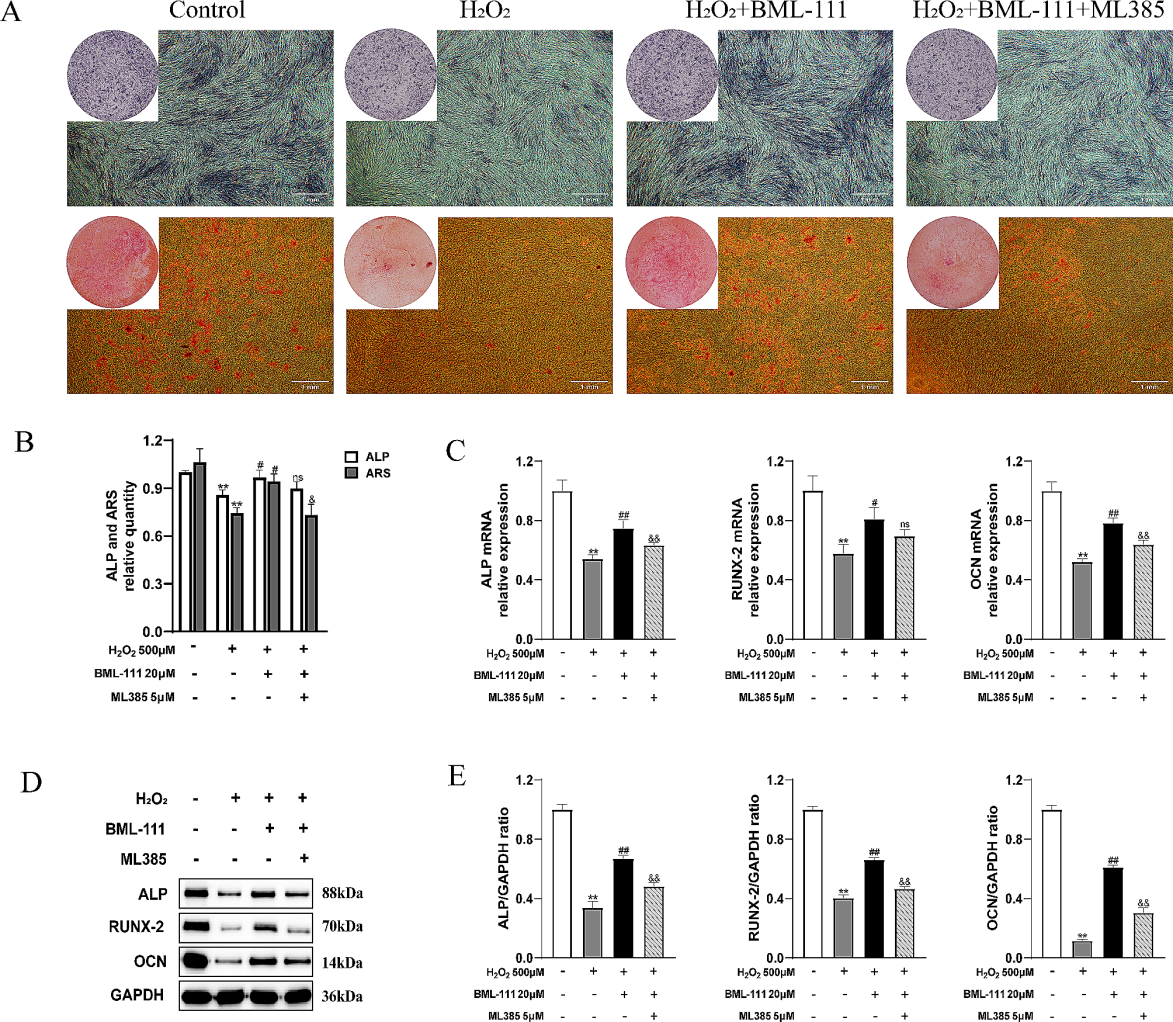
ML385 inhibit the effect of BML-111 on the H_2_O_2_-induced osteogenic dysfunction in hPDLFs. **(A)** Macroscopic and Microscopic images of ALP Staining and Alizarin Red Staining. **(B)** The ALP and ARS staining quantification of hPDLFs was shown. **(C)** The ALP, RUNX-2, OCN mRNA relative expression was shown. **(D)** Representative Western blots for ALP, RUNX-2, OCN protein. **(E)** The ALP, RUNX-2, OCN protein relative expression was shown. ^**^P < 0.01 compared with control group. ^##^*P* < 0.01 compared with H_2_O_2_ group. ^&&^*P* < 0.01 compared with H_2_O_2_ + BML-111 group. ^ns^*P*>0.05 compared with control group. ^#^*P* < 0.05 compared with H_2_O_2_ group. ^&^*P* < 0.05 compared with H_2_O_2_ + BML-111 group. ^ns^*P*>0.05 compared with BML-111 + H_2_O_2_ group

As shown in Fig. 7A, in the H_2_O_2_ group, the expression of ALP and the generation of calcium nodules significantly decreased compared with the control group, as well as the expressions of ALP, RUNX-2, and OCN genes and proteins. BML-111can effectively alleviated the inhibition of the ALP, RUNX-2, OCN expression in hPDLFs caused by H_2_O_2_ (Fig. 7B, C, D and E). The addition of ML385 inhibited the protective effect of BML-111 on H_2_O_2_-induced osteogenic dysfunction.

## Discussion

This study is the first to demonstrate that BML-111 can inhibit the activation of NLRP3 inflammasome related-pyroptosis and osteogenic dysfunction in hPDLFs under oxidative stress by activating the Nrf2/HO-1 signaling pathway. Therefore, BML-111, as an endogenous anti-inflammatory mediator analog, provide a new idea for the prevention and treatment of periodontitis.

Periodontitis is a chronic inflammatory disease, and the key to its pathogenesis lies in the host’s immune response to pathogenic bacteria. With the consistently stimulation of the pathogenic bacteria and its toxicity factors, periodontal tissues overproduce ROS, which leads to an oxidative imbalance that triggers proinflammatory mechanisms [21]. The overproduction of ROS by hPDLFs is the key signal in the inflammatory response because it can activate the NLRP3 inflammasome, leading to the destruction of periodontal soft and hard tissues [22]. According to the study, mice with periodontitis exhibit an increase in NLRP3 inflammasome expression, and inhibit the activation of the NLRP3 inflammasome will alleviate the occurrence of periodontitis [7, 23]. The main mechanism of the pro-inflammatory effect of NLRP3 inflammasome is to promote the conversion of pro- Caspase-1 to active Caspase-1, which in turn promotes the maturation and secretion of IL-1β and IL-18. At the same time, activated Caspase-1 leads to cleavage of GSDMD protein to generate GSDMD-N fragment. GSDMD-N oligomerizes and forms pores in the cell membrane, leading to the occurrence of pyroptosis [24]. During pyroptosis, cells will swell, perforate, lyse, and eventually release their contents as a result of pro-inflammatory programmed cell death. Cellular integrity is destroyed during this process, and cellular contents are released into the extracellular space, promoting inflammation [25]. In this study, it was found that the application of H_2_O_2_ resulted in increased intracellular ROS in hPDLFs, which in turn activated the NLRP3 inflammasome. In this study, it was confirmed that the application of H_2_O_2_ led to increased cell death in hPDLFs, accompanied by a large amount release of IL-1β and IL-18, the mechanism was that excessive ROS activated NLRP3 inflammasome and finally caused hPDLFs pyroptosis.

The occurrence of pyroptosis leads to the release of IL1β and IL-18, which makes hPDLFs in an inflammatory microenvironment and further aggravates cell dysfunction [26]. Studies have shown that activation of NLRP3 inflammasome in periodontal and gingival cells can induce pyroptosis, leading to the destruction of alveolar bone. Pyroptosis leads to IL-1β activation, induction of RANKL production and enhancement of osteoclast activity [27]. At the same time, IL-18 can promote the level of matrix metalloproteinase protein in periodontal ligament cells and increase the production of osteoclasts [28]. Caspase-1 were highly expressed in the periodontal tissues of experimental periodontitis rats, and the alveolar bone destruction of periodontitis rats was alleviated after the application of Caspase-1 inhibitors, respectively, indicating that the pyroptosis plays an important role in bone resorption in periodontitis [29]. In a rat model of periodontitis, drug inhibition of Caspase-4 or IL-1β antibody significantly reduced alveolar bone loss and periodontal soft tissue injury [19, 30, 31]. The results of this study showed that H_2_O_2_ could lead to NLRP3 inflammatory-related pyroptosis of cells, followed by the release of inflammatory factors, impaired osteogenic differentiation ability of cells, and down-regulated expression of osteoblast-related proteins and genes. BML-111 inhibited pyroptosis induced by H_2_O_2_, thus alleviating the osteogenic differentiation injury of cells.

LXA4 is an endogenous anti-inflammatory mediator that acts as an inflammatory brake signal in the process of inflammation. It plays an important role in the pathological outcome of inflammatory processes such as ischemia reperfusion injury, inhibitory epidermitis, peritonitis, colitis, gastroenteritis, asthma and keratitis [19, 32, 33]. The presence of LXA4 in the gingival crevicular fluid of patients with active periodontitis suggests a potential role for this immunomodulatory molecule in the development and outcome of local inflammation in the periodontal tissue [34]. LXA4 is a drug with few side effects because it is rapidly inactivated in the body, but it has a short half-life and its efficacy is not very durable. BML-111 is a relatively stable and potent LXA4 analog, which has the same effect as LXA4 in terms of anti-inflammatory effect [20]. BML-111 has previously been shown to be comparable to LXA4 in suppressing inflammatory processes. This agonist has been shown to promote catabolism and anti-inflammatory effects in acute lung injury, collagenous arthritis, and acute pancreatitis [33, 35, 36]. This study confirmed that BML-111 can inhibit the pyroptosis of hPDLFs caused by H_2_O_2_, thereby inhibiting the inflammatory response and reducing the damage of osteogenic differentiation capacity. The beneficial effect of BML-111 on inflammation is achieved by activating the Nrf2/HO-1 signal pathway. Pretreatment of hPDLFs with BML-111 can increase the expression of intracellular Nrf2, promote its translocation into the nucleus, activate the ARE antioxidant elements, promote the expression of downstream antioxidant enzymes HO-1, which can improve the antioxidant capacity of cells, thereby preventing excessive accumulation of ROS in cells, reducing the occurrence of pyroptosis and the release of inflammatory mediators. Much literature have shown that Nrf2-Keap1 complex is located in cytoplasm under static condition and degraded by ubiquitination and proteolytic hydrolysis, which maintains a low Nrf2 expression level [37]. While under stress environment, Nrf2-Keap1 complex dissociates and releases Nrf2, and the free Nrf2 is transferred to the nucleus. Therefore, promoting the dissociation of Nrf2-Keap1 complex is conducive to the activation and up-regulation of Nrf2 expression. The ways of promoting Nrf2-Keap1 dissociation include changing the conformation of Keap1, Nrf2 phosphorylation and competitive binding of Keap1. Recent studies have shown that autophagy related protein P62 can enhance the expression of Nrf2 by competitively binding Keap1 and promoting the autophagy degradation of Keap1, and the activation of Nrf2 further up-regulates the expression of P62, forming a positive feedback cycle [38, 39]. We observed that BML-111 promoted the activation of P62 which can competing binding with Keap1 and up-regulates the expression of Nrf2. In addition, As an inhibitor of Nrf2, ML385 inhibits the translocation of Nrf2 into the nucleus and the binding of the antioxidant element ARE, reducing the transcriptional activity of Nrf2 [40]. ML385 also reduced the activity of the Nrf2 promoter, resulting in reduced expression of Nrf2 [41]. In our study, we inhibited Nrf2 with ML385 and then treated cells with BML-111. Our findings suggest that BML-111 cannot attenuate H_2_O_2_-induced oxidative damage if Nrf2 is inhibited, and these findings support the notion that BML-111 protects hPDLFs primarily through the Nrf2/HO-1 pathway.

## Conclusion

Taken together, our study clarified that BML-111 may attenuate H_2_O_2_-induced hPDLFs dysfunction through the Nrf2/HO-1 signaling pathway. Excessive intracellular ROS induces NLRP3 inflammasome activation and cell pyroptosis, resulting in osteogenic dysfunction of hPDLFs. By activating the Nrf2/HO-1 pathway, BML-111 reduces intracellular ROS and alleviates the dysfunction of hPDLFs. This study validates the anti-periodontitis effect of BML-111 and proposes a possible clinical treatment approach for periodontitis by exogenous supplementation of endogenous anti-inflammatory mediator analogs.

### Electronic supplementary material

Below is the link to the electronic supplementary material.

Supplementary Material 1

## Data Availability

The datasets used and/or analyzed during the current study are available from corresponding author on reasonable request.

## References

[1] Hajishengallis G (2015). Periodontitis: from microbial immune subversion to systemic inflammation. Nat Rev Immunol.

[2] Symmank J, Chorus M, Appel S, Marciniak J, Knaup I, Bastian A, Hennig CL, Döding A, Schulze-Späte U, Jacobs C (2020). Distinguish fatty acids impact survival, differentiation and cellular function of periodontal ligament fibroblasts. Sci Rep.

[3] Chu Y, Xu Y, Yang W, Chu K, Li S, Guo L (2023). N-acetylcysteine protects human periodontal ligament fibroblasts from pyroptosis and osteogenic differentiation dysfunction through the SIRT1/NF-κB/Caspase-1 signaling pathway. Arch Oral Biol.

[4] Aral K, Milward MR, Kapila Y, Berdeli A, Cooper PR (2020). Inflammasomes and their regulation in periodontal Disease: a review. J Periodontal Res.

[5] Abais JM, Xia M, Zhang Y, Boini KM, Li PL (2015). Redox regulation of NLRP3 inflammasomes: ROS as trigger or effector?. Antioxid Redox Signal.

[6] Abderrazak A, Syrovets T, Couchie D, El Hadri K, Friguet B, Simmet T, Rouis M (2015). NLRP3 inflammasome: from a danger signal sensor to a regulatory node of oxidative stress and inflammatory Diseases. Redox Biol.

[7] Chen Q, Liu X, Wang D, Zheng J, Chen L, Xie Q, Liu X, Niu S, Qu G, Lan J (2021). Periodontal inflammation-triggered by Periodontal Ligament Stem Cell Pyroptosis exacerbates Periodontitis. Front cell Dev Biology.

[8] Lian D, Dai L, Xie Z, Zhou X, Liu X, Zhang Y, Huang Y, Chen Y (2018). Periodontal ligament fibroblasts migration injury via ROS/TXNIP/Nlrp3 inflammasome pathway with Porphyromonas gingivalis lipopolysaccharide. Mol Immunol.

[9] Badibostan H, Eizadi-Mood N, Hayes AW, Karimi G. Protective effects of natural compounds against paraquat-induced pulmonary toxicity: the role of the Nrf2/ARE signaling pathway. Int J Environ Health Res 2023:1–14. 10.1080/09603123.2022.2163985 36682065

[10] Schieffer L, Manzl C, Schatz C, Haybaeck J, Crismani A. Nrf2 in the field of Dentistry with Special attention to NLRP3. Antioxid (Basel) 2022, 11(1). 10.3390/antiox11010149 PMC8772975 35052653

[11] Arioz BI, Tastan B, Tarakcioglu E, Tufekci KU, Olcum M, Ersoy N, Bagriyanik A, Genc K, Genc S (2019). Melatonin attenuates LPS-Induced Acute Depressive-Like behaviors and Microglial NLRP3 inflammasome activation through the SIRT1/Nrf2 pathway. Front Immunol.

[12] Huang C, Zhang C, Yang P, Chao R, Yue Z, Li C, Guo J, Li M (2020). Eldecalcitol inhibits LPS-Induced NLRP3 inflammasome-dependent pyroptosis in human gingival fibroblasts by activating the Nrf2/HO-1 signaling pathway. Drug Des Devel Ther.

[13] Das UN. Essential fatty acids and their metabolites in the pathobiology of inflammation and its resolution. Biomolecules 2021, 11(12). 10.3390/biom11121873 PMC8699107 34944517

[14] Wu L, Li HH, Wu Q, Miao S, Liu ZJ, Wu P, Ye DY (2015). Lipoxin A4 activates Nrf2 pathway and ameliorates cell damage in cultured cortical astrocytes exposed to oxygen-glucose Deprivation/Reperfusion insults. J Mol Neuroscience: MN.

[15] Ali M, Kucko N, Jansen JA, Yang F, Walboomers XF (2021). The effect of lipoxin A4 on E. Coli LPS-induced osteoclastogenesis. Clin Oral Investig.

[16] Ali M, Yang F, Jansen JA, Walboomers XF (2020). Lipoxin suppresses inflammation via the TLR4/MyD88/NF-kappaB pathway in periodontal ligament cells. Oral Dis.

[17] Almudena V-B, Patricia P, Iñigo JR, Marta G-F, Marta P, Nieves D, Verónica T, María T, Inmaculada J, Jesús V et al. Specialized Proresolving mediators protect against experimental autoimmune Myocarditis by modulating Ca2 + handling and NRF2 activation %J JACC: Basic to Translational Science. 2022, 7(6). 10.1016/j.jacbts.2022.01.009 PMC9270570 35818504

[18] Hasturk H, Schulte F, Martins M, Sherzai H, Floros C, Cugini M, Chiu CJ, Hardt M, Van Dyke T (2021). Safety and preliminary efficacy of a Novel host-modulatory therapy for reducing Gingival inflammation. Front Immunol.

[19] Shi Z, Wang Y, Ye W, Lin Z, Deng T, Zhang T, Zhao J, Tong Y, Shan Y, Chen G (2021). The LipoxinA4 receptor agonist BML-111 ameliorates intestinal disruption following acute Pancreatitis through the Nrf2-regulated antioxidant pathway. Free Radic Biol Med.

[20] Fen X, Jiamin Z, Xiaoyan Z, Hua H. Lipoxin A4 and its analog attenuate high fat diet-induced Atherosclerosis via Keap1/Nrf2 pathway %J. Exp Cell Res. 2022(prepublish). 10.1016/j.yexcr.2022.113025 35026282

[21] Sczepanik FSC, Grossi ML, Casati M, Goldberg M, Glogauer M, Fine N, Tenenbaum HC (2020). Periodontitis is an inflammatory Disease of oxidative stress: we should treat it that way. Periodontol 2000.

[22] Liu S, Du J, Li D, Yang P, Kou Y, Li C, Zhou Q, Lu Y, Hasegawa T, Li M (2020). Oxidative stress induced pyroptosis leads to osteogenic dysfunction of MG63 cells. J Mol Histol.

[23] Yu C, Zhang C, Kuang Z, Zheng Q (2021). The role of NLRP3 inflammasome activities in Bone Diseases and vascular calcification. Inflammation.

[24] Li Y, Li B, Liu Y, Wang H, He M, Liu Y, Sun Y, Meng W (2021). Porphyromonas gingivalis lipopolysaccharide affects oral epithelial connections via pyroptosis. J Dent Sci.

[25] Frank D, Vince JE (2019). Pyroptosis versus necroptosis: similarities, differences, and crosstalk. Cell Death Differ.

[26] Ebe Y, Nakamura T, Hasegawa-Nakamura K, Noguchi K (2021). Effect of interleukin-1β on bone morphogenetic protein-9-induced osteoblastic differentiation of human periodontal ligament fibroblasts. Eur J Oral Sci.

[27] Alam MI, Mae M, Farhana F, Oohira M, Yamashita Y, Ozaki Y, Sakai E, Yoshimura A. NLRP3 Inflammasome negatively regulates RANKL-Induced Osteoclastogenesis of Mouse Bone Marrow macrophages but positively regulates it in the Presence of Lipopolysaccharides. Int J Mol Sci 2022, 23(11). 10.3390/ijms23116096 PMC9181162 35682777

[28] Wang F, Guan M, Wei L, Yan H (2019). IL–18 promotes the secretion of matrix metalloproteinases in human periodontal ligament fibroblasts by activating NF–κB signaling. Mol Med Rep.

[29] Jiang M, Shang Z, Zhang T, Yin X, Liang X, Sun H (2022). Study on the role of pyroptosis in bone resorption induced by occlusal trauma with or without periodontitis. J Periodontal Res.

[30] Zhang R, Wu Z, Li M, Yang J, Cheng R, Hu T (2022). Canonical and noncanonical pyroptosis are both activated in periodontal inflammation and bone resorption. J Periodontal Res.

[31] Liu X, Wang X, Duan X, Poorun D, Xu J, Zhang S, Gan L, He M, Zhu K, Ming Z (2017). Lipoxin A4 and its analog suppress inflammation by modulating HMGB1 translocation and expression in psoriasis. Sci Rep.

[32] Futokoro R, Hijioka M, Arata M, Kitamura Y. Lipoxin A(4) receptor stimulation attenuates Neuroinflammation in a mouse model of Intracerebral Hemorrhage. Brain Sci 2022, 12(2). 10.3390/brainsci12020162 PMC8869920 35203926

[33] Xu F, Zhang J, Zhou X, Hao H (2022). Lipoxin A4 and its analog attenuate high fat diet-induced Atherosclerosis via Keap1/Nrf2 pathway. Exp Cell Res.

[34] Cianci E, Recchiuti A, Trubiani O, Diomede F, Marchisio M, Miscia S, Colas RA, Dalli J, Serhan CN, Romano M (2016). Human Periodontal stem cells release Specialized Proresolving mediators and carry Immunomodulatory and Prohealing Properties regulated by Lipoxins. Stem Cells Transl Med.

[35] Wang L, Su W, Zheng X, Lin W, Lv C, Yang S, Chen B, Zhang C (2023). BML-111 inhibits osteoclast differentiation by suppressing the MAPK and NF-κB pathways, alleviating deterioration of the knee joints in a CIA rat model. Cell Biol Int.

[36] Zou F, Zhuang ZB, Zou SS, Wang B, Zhang ZH (2022). BML-111 alleviates inflammatory response of alveolar epithelial cells via miR-494/Slit2/Robo4 signalling axis to improve acute lung injury. Autoimmunity.

[37] Crisman E, Duarte P, Dauden E, Cuadrado A, Rodríguez-Franco MI, López MG, León R (2023). KEAP1-NRF2 protein-protein interaction inhibitors: design, pharmacological properties and therapeutic potential. Med Res Rev.

[38] Jain A, Lamark T, Sjøttem E, Larsen KB, Awuh JA, Øvervatn A, McMahon M, Hayes JD, Johansen T (2010). p62/SQSTM1 is a target gene for transcription factor NRF2 and creates a positive feedback loop by inducing antioxidant response element-driven gene transcription. J Biol Chem.

[39] Jiang T, Harder B, Rojo de la Vega M, Wong PK, Chapman E, Zhang DD (2015). p62 links autophagy and Nrf2 signaling. Free Radic Biol Med.

[40] Xian P, Hei Y, Wang R, Wang T, Yang J, Li J, Di Z, Liu Z, Baskys A, Liu W (2019). Mesenchymal stem cell-derived exosomes as a nanotherapeutic agent for amelioration of inflammation-induced astrocyte alterations in mice. Theranostics.

[41] Singh A, Venkannagari S, Oh KH, Zhang YQ, Rohde JM, Liu L, Nimmagadda S, Sudini K, Brimacombe KR, Gajghate S (2016). Small molecule inhibitor of NRF2 selectively intervenes therapeutic resistance in KEAP1-Deficient NSCLC tumors. ACS Chem Biol.

