# Supplementary material for: BML-111 inhibit H2O2-induced pyroptosis and osteogenic dysfunction of human periodontal ligament fibroblasts by activating the Nrf2/HO-1 pathway
Source: BMC Oral Health. 2024 Jan 8;24:40. doi: 10.1186/s12903-023-03827-w (PMC10773113; doi:10.1186/s12903-023-03827-w)
Supplement: Supplementary file 1 — Supplementary Material 1 [file 12903_2023_3827_MOESM1_ESM.pdf]

Figure 5 ASC

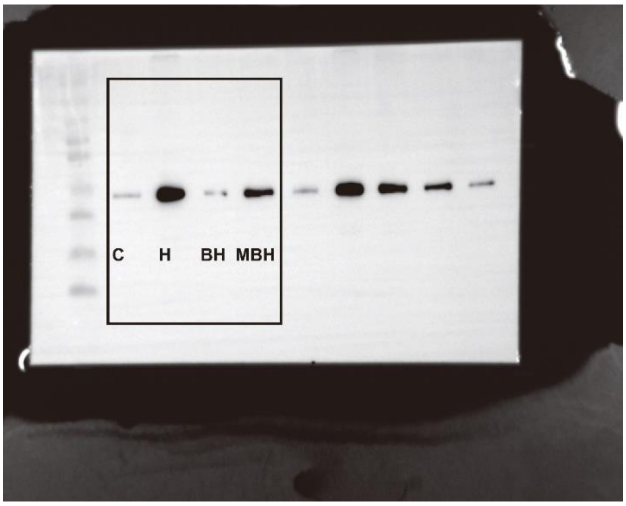

Figure 5 Caspase 1

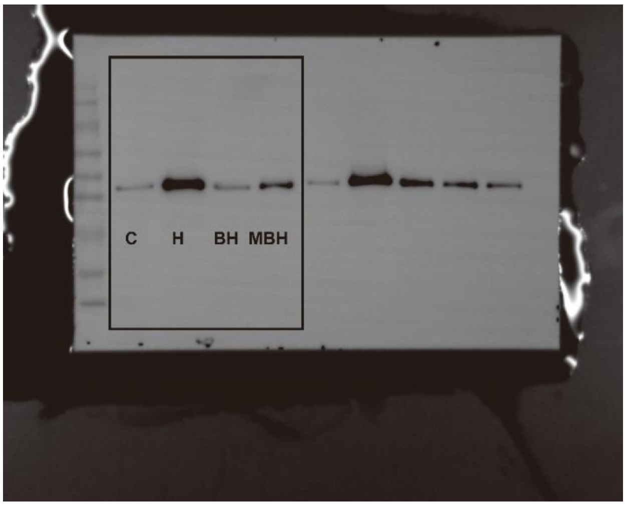

Figure 5 GAPDH

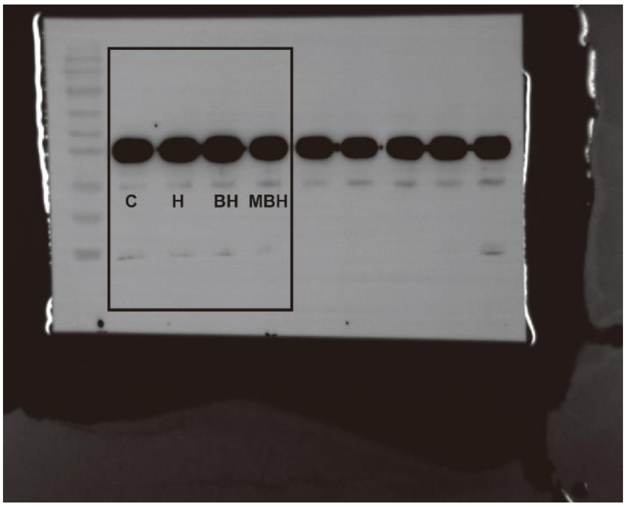

Figure 5 GSDMD-N

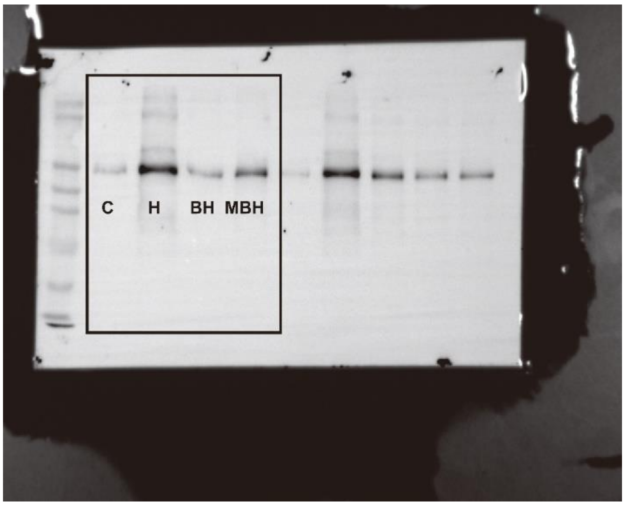

Figure 5 HO-1

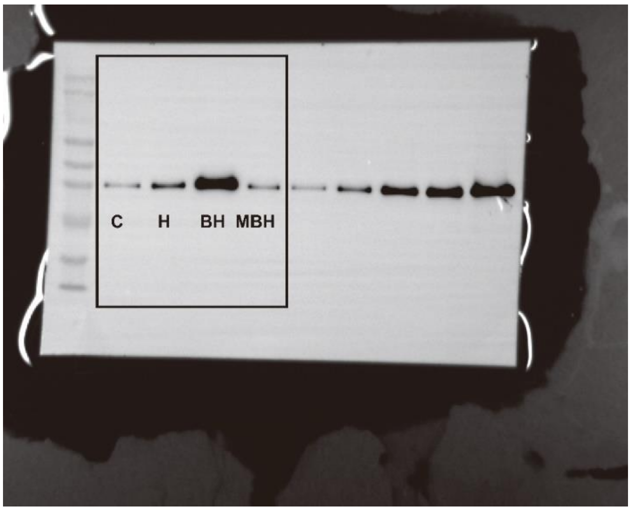

Figure 5 Lamin

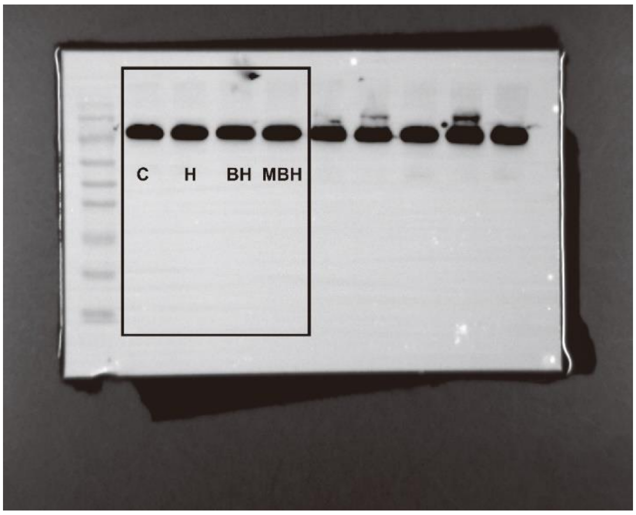

Figure 5 NLRP3

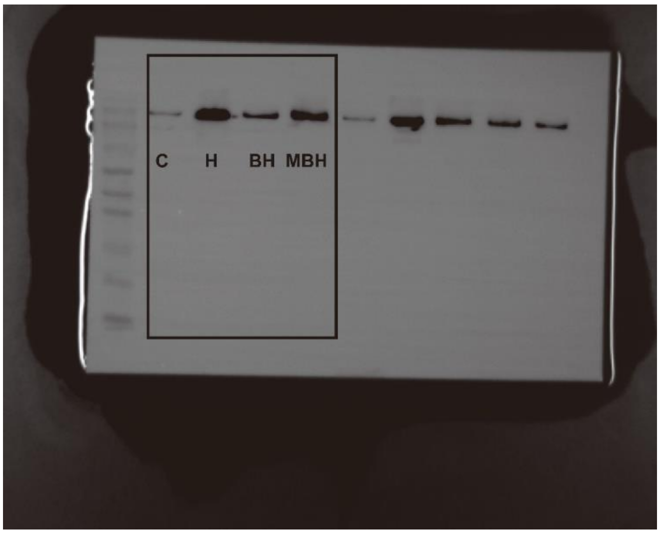

Figure 5 Nrf2

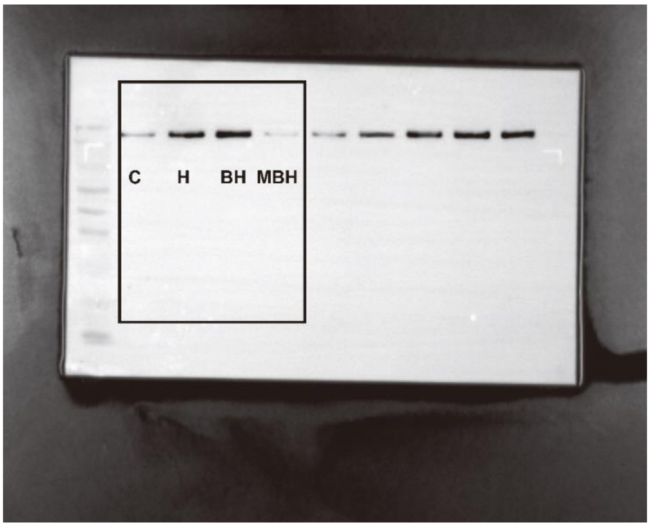

Figure 5 Nrf2-nuclear

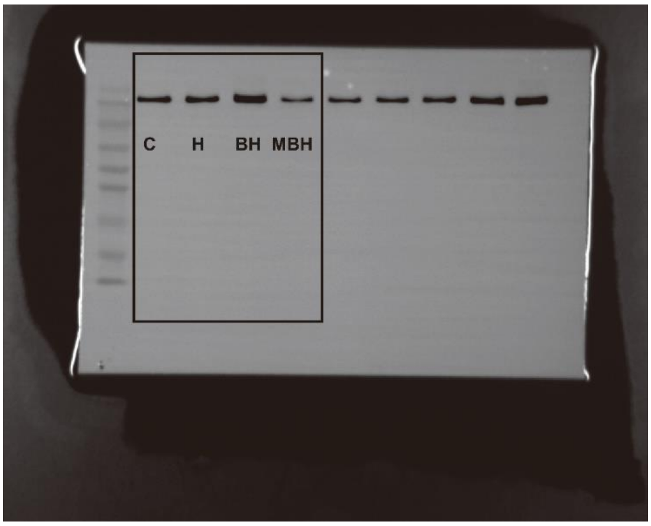

Figure 6 Keap1

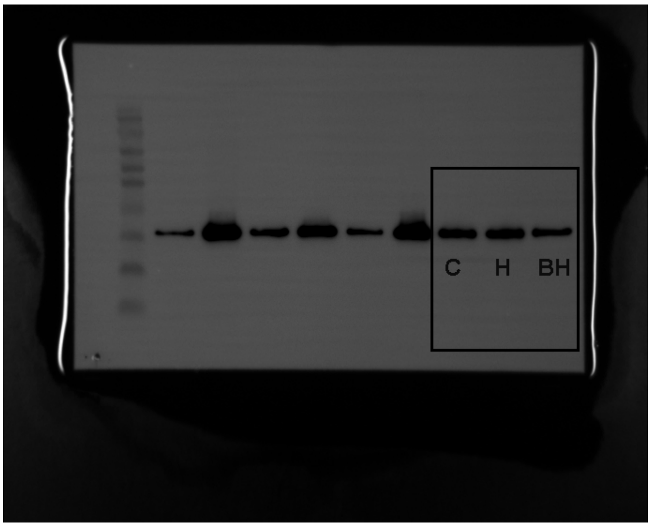

Figure 6 P62

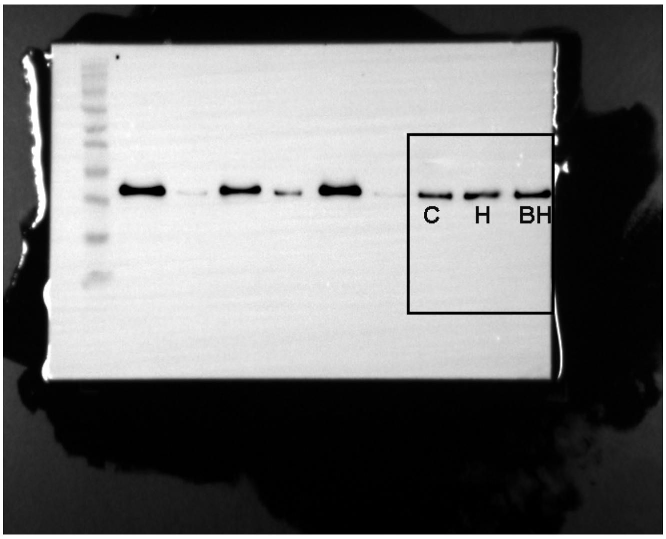

Figure 6 GAPDH

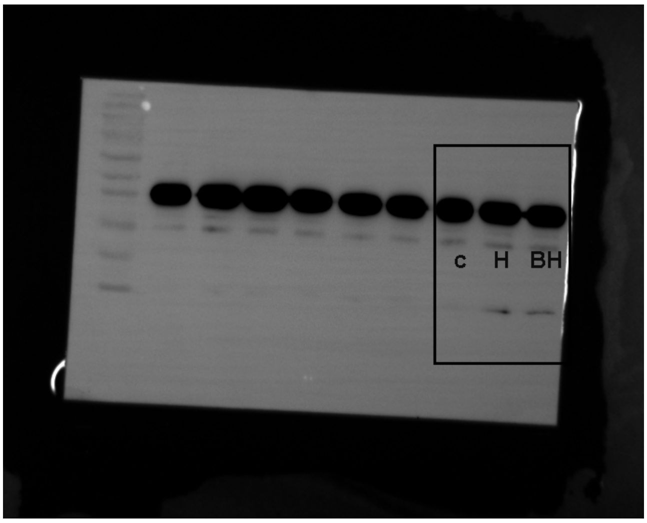

Figure 7ALP

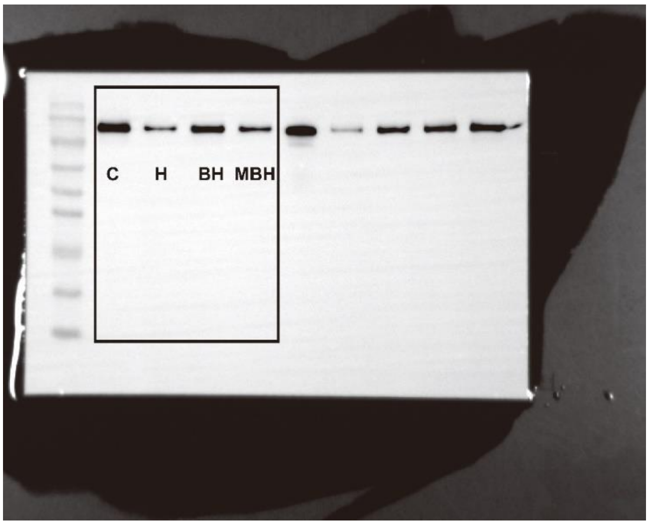

Figure 7 OCN

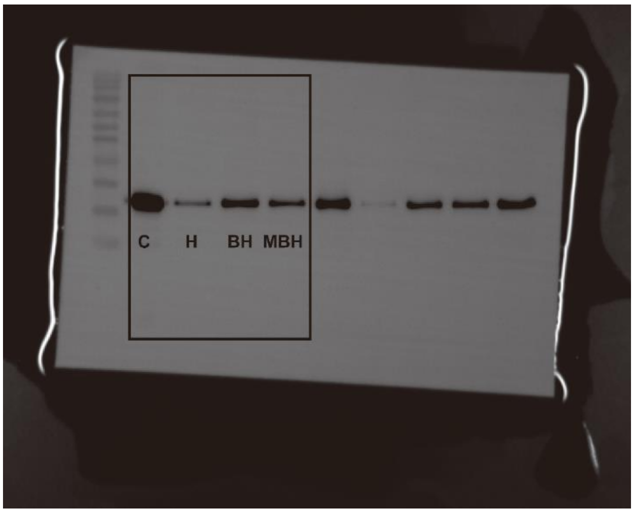

Figure 7 RUNX-2

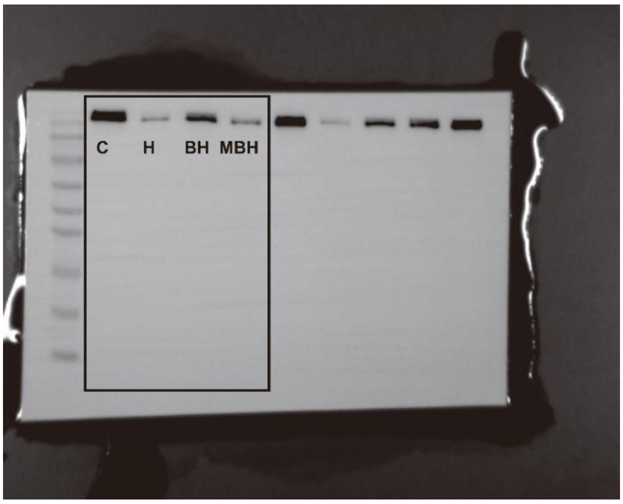

Figure 7 GAPDH

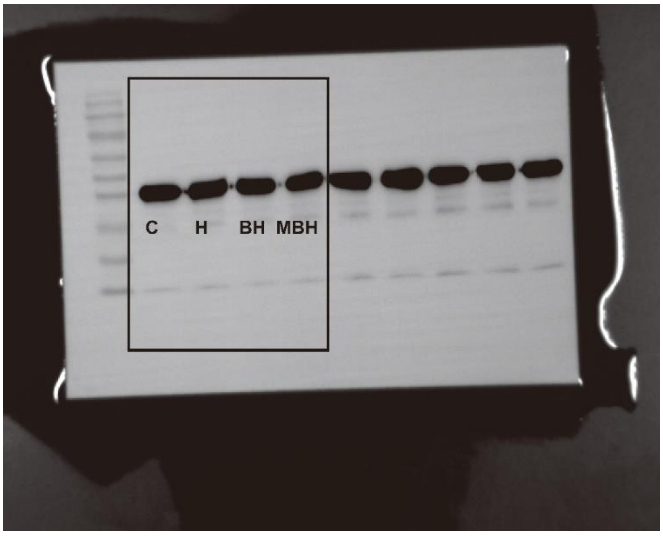

C: control group

H: H_2O_2 group

BH: BML-111+ H_2O_2 group

MBH: ML385+ BML-111+ H_2O_2 group
